# Melt Memory Effect in Polyethylene Random Terpolymer with Small Amount of 1-Octene and 1-Hexene Co-Units: Non-Isothermal and Isothermal Investigations

**DOI:** 10.3390/polym15071721

**Published:** 2023-03-30

**Authors:** Dengfei Wang, Shiyan Li, Ying Lu, Jian Wang, Yongfeng Men

**Affiliations:** 1 Provincial Key Laboratory of Oil & Gas Chemical Technology, College of Chemistry & Chemical Engineering, Northeast Petroleum University, Daqing 163318, China; 2 Daqing Petrochemical Research Center, Petrochemical Research Institute of PetroChina, Daqing 163714, China; 3 State Key Laboratory of Polymer Physics and Chemistry, Changchun Institute of Applied Chemistry, Chinese Academy of Sciences, Renmin Street 5625, Changchun 130022, China

**Keywords:** polyethylene copolymer, thermal analysis, crystallization, memory effect

## Abstract

Homo-polymers of reasonable molecular weight relax very fast in the molten state. Starting from a semi-crystalline structure, when the homo-polymer is heated up to a temperature higher than its nominal melting temperature, it relaxes quickly into a homogenous molten state. The following crystallization temperature during cooling remains constant irrespective of the melt temperature. However, the situation is evidently different in copolymers. A phenomenon named the crystallization melt memory effect denotes an increased crystallization rate during cooling after a polymer was melted at different temperatures, which is often observed. The melt temperature can be even higher than the equilibrium melting temperature of the corresponding polymer crystals. In this work, we investigated such memory effect in a polyethylene random terpolymer with a small fraction of 1-octene and 1-hexene co-units using differential scanning calorimetry techniques. Both non-isothermal and isothermal protocols were employed. In non-isothermal tests, a purposely prepared sample with well defined thermal history (the sample has been first conditioned at 200 °C for 5 min to eliminate the thermal history and then cooled down to −50 °C) was melted at different temperatures, followed by a continuous cooling at a constant rate of 20 °C/min. Peak crystallization temperature during cooling was taken to represent the crystallization rate. Whereas, in isothermal tests, the same prepared sample with well defined thermal history was cooled to a certain crystallization temperature after being melted at different temperatures. Here, time to complete the isothermal crystallization was recorded. It was found that the results of isothermal tests allowed us to divide the melt temperature into four zones where the features of the crystallization half time change.

## 1. Introduction

As a typical semi-crystalline polymer, polyethylene possesses a flexible chain structure and is easy to crystallize into a rather high crystallinity, resulting in a strong and tough material at room temperature. The addition of a small amount of non-crystallizable co-units randomly onto the polyethylene main chain can alter the crystallization of polyethylene chain segments in a way that both the crystallization temperature and the crystallinity decrease. Copolymerization of a second or even more different kinds of co-monomers with ethylene can tune the properties of the copolymers effectively [1,2,3,4,5,6,7,8,9,10]. Typical examples are ethylene-1-alkene copolymers, which are widely used in many different fields, such as pipes for water, oil, and gas transportation, packing films for foods and goods, structural materials for furniture or containers, and so on [1,7] These varieties of applications require a wide range of mechanical properties of the final polyethylene products. The presence of a chain of co-units at random positions, stereo defects, or noncrystallizable second polymers in the melt cannot be included in the crystal lattice, but it affects the crystallization and melting properties [1,2,3,4,5,6,7,8,9,10,11,12,13,14,15,16,17,18,19,20,21,22,23,24,25,26,27,28,29].

Based on the assumption of the pure crystalline phase, the relationship between the equilibrium melting temperature, $T_{m}$, and the comonomer content of the copolymer has been developed by Flory [2] in the 1950s. For a random copolymer, there is:

(1) $$\begin{array}{c} 1/T_{m}-1/T_{m}^{0}=-\left(R/{∆H}_{u}\right)\ln\left(1-X_{A}\right) \end{array}$$

where $T_{m}^{0}$ is the equilibrium melting temperature of the pure homopolymer, *R* is the gas constant, ${∆H}_{u}$ is the heat of fusion per mole of chain units, and $X_{A}$ is the mole fraction of crystallizable units. According to Equation (1), the specific chemical composition of the non-crystallizable part will not affect the theoretical prediction results. A major shortcoming of the theory is that the value of ${∆H}_{u}$ obtained for real systems is much lower than other theoretical methods [7] Besides, there exists a systematic difference between the observed melting temperature and the equilibrium theoretical temperature. This is because, on the one hand, the melting temperature represents the disappearance of the crystallite. However, for copolymers in which the crystallization of the sequences will be restricted, the detection requires extremely sensitive experimental techniques [3,4]. On the other hand, the average crystallite thickness is less than the equilibrium requirements and decreases with the increase in the co-unit content [8].

The crystallization process of copolymers is often accompanied by the melt memory effect, which is correlated with self-seeds that increase the crystallization rate of copolymers [27,28,30]. If a copolymer is melted and then crystallized again, the time required for the subsequent crystallization process often changes with the previous melt temperature and the duration of the molten stage [1,11,16,17,19,22,23,27,28,29]. The parameters, such as comonomer content, distribution, and melt temperature reached, will affect the strength and tendency of the melt memory effect [16,18,19].

The memory effect below the equilibrium melting point can also be classified as the self-seeding nucleation effect, which is due to the presence of partial micro-crystallites in the sample that is not completely molten [16,25,26,27]. Poly(ethylene-co-octene) analyzed by Strobl et al. [11], with the equilibrium melting temperature of 132.1 °C, showed the crystallization kinetics varies only between 120 and 127 °C, which is well below the equilibrium melting temperature for this copolymer. Polymer synthesis often relies on catalysts, granulators, emulsifiers, etc. to obtain commercial materials with wide distribution or chemical modification. These additives or residues may act as nucleating agents during the crystallization of the polymer melt [18]. The additives are the heterogeneous nucleus and not resolved above the equilibrium melting point [18,20]. Liu et al. [25] reported such a case, where the melt memory effect above the equilibrium melting point disappeared after purifying the commercial polybutene-1 sample. The results reveal that nuclei derived from external additives cause melt memory effects in homopolymers.

When self-nuclei exist at a temperature higher than the equilibrium melting point, the melts will show a “strong melt memory of the crystallization” [1,23,24,25,30,31]. Alamo et al. [23] reported the effect of molecular weight and comonomer content on melt crystallization of model random ethylene/1-butene copolymers. The crystallization temperature of unbranched liner PE samples was constant, and no significant memory effect was shown, and copolymers with molar mass below 4500 g/mol had the same independence. The strength of melt memory is molar mass-dependent and decreases, with comonomer content increasing, and it vanishes at 4.53 mol% branches. It can be explained that the amorphous region of the highly branched copolymer is less constrained, which allows crystalline sequences to diffuse more readily. By means of dynamic Monte Carlo simulations of a random copolymer, Hu et al. [1] attributed the effect to the locally high concentration of long sequences after previous crystallization. This strong melt memory means that the de-mixing of different sequences in random copolymers affects the crystallization.

In this work, we investigated the crystallization behavior of a newly synthesized polyethylene random terpolymer with a small fraction of 1-octene and 1-hexene (in total 1.1 mol%). It turned out that this polyethylene random terpolymer exhibits strong melt memory effect after being melted at different temperatures. Moreover, isothermal crystallization investigations clearly divided the melt temperature into four zones, where the trend of the kinetics of the followed crystallization changes.

## 2. Experimental Section

### 2.1. Materials

**Scheme 1:**
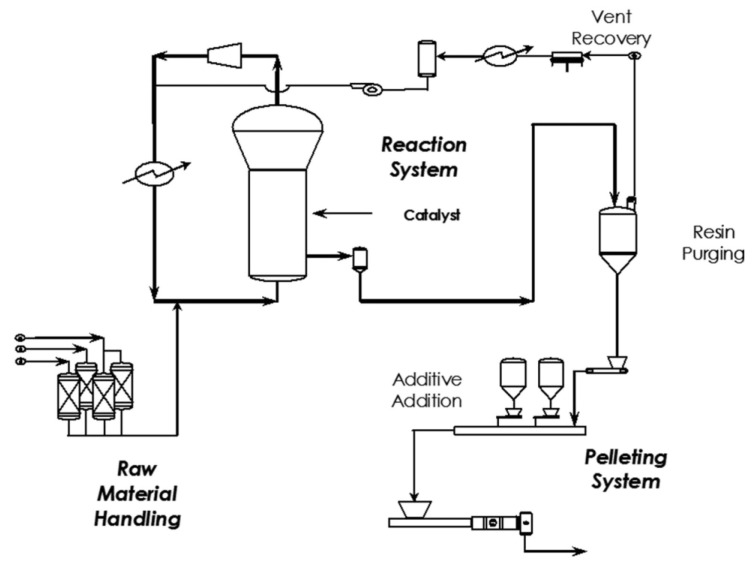
Schematic representation of the preparation process of the ethylene/1-octene/1-hexene random terpolymer.

The sample used in this study was an ethylene/1-octene/1-hexene random terpolymer (P(E-co-O-co-H)) prepared by metallocene catalyst as follows. Fresh ethylene and co-monomer, 1-octene, and 1-hexene are mixed in a fixed ratio, and they are fed to a 50 kg/h polyethylene pilot plant that is passed through a separate series of purifiers where trace quantities of impurities are removed. The ethylene and co-monomers are fed to the reaction system. The reaction system consists of a fluid bed reactor, a cycle gas compressor and cooler, and product discharge tanks. Ethylene, co-monomers, and a recycle stream from the vent recovery system are fed continuously to the reactor. P(E-co-O-co-H) is removed from the reactor by the discharge tanks and sent to a purge tank where unreacted monomer and dissolved hydrocarbons are stripped from the resin and are sent to the vent recovery system. The purged ethylene/1-octene/1-hexene copolymer is sent to the pelleting system. The vent recovery system recovers as many hydrocarbons as possible from the streams sent to it. The condensed components are returned directly to the reaction system, and the light gases are used as conveying gases to reduce nitrogen consumption. Solid additives are metered and sent to the pelleting system. The resin, solid additives, and liquid additives are mixed, melted, and pelleted in the pelleting system. The pellets were dried, cooled, and sent to product blending and storage. The procedure of the polymer preparation is given in Scheme 1 below.

The weight-average molecular weight (M_w_) of the copolymer is 106,600 g/mol, and the number-average molecular weight (M_n_) is 41,700 g/mol, as measured by gel permeation chromatography (GPC) listed in Table 1. The ethylene content is about 98.9%, and the comonomers are about 1.1 mol%. Additionally, listed are crystallinity and peak melting temperatures obtained from differential scanning calorimetry after cooling and heating at 10 °C/min. 

### 2.2. DSC Tests

DSC measurements were performed using a DSC1 Star^e^ system (Mettler Toledo Instruments, Switzerland) under a nitrogen atmosphere (50 mL/min). The DSC instrument had been calibrated using indium as a standard before all the measurements.

Pellets of P(E-co-O-co-H) terpolymer were first melted at 180 °C for 5 min to develop a thin film with a thickness of 0.5 mm and then held at this temperature for 10 min under a pressure of 20 MPa. Three min were left to allow polymer chains to relax at 180 °C after removing the pressure. After that, the hot sheets were quickly transferred to room temperature and kept for more than 1 h. The samples used in each experiment were cut from the thin film, and the sample weight was about 6 mg. 

## 3. Results and Discussion

**Figure 1:**
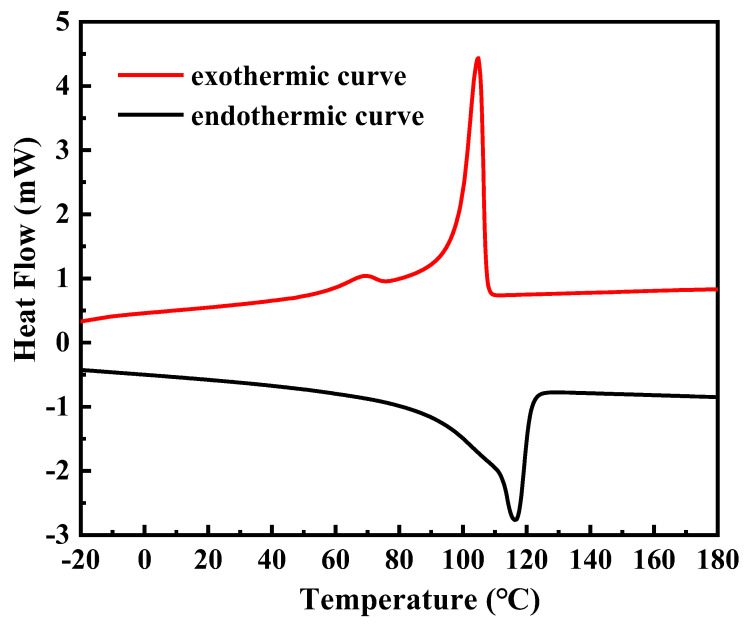
DSC cooling and heating curves of P(E-co-O-co-H) terpolymer with the speed of 10 °C/min. The endothermic peak at melting is downward and the exothermic peak at cooling is upward.

Figure 1 presents the DSC curves of the P(E-co-O-co-H) terpolymer during cooling from 180 °C to −20 °C, followed by a heating scan to 180 °C. During cooling, one observes a strong and narrow exothermic peak, starting from about 110 °C, and a much weaker peak at around 70 °C, indicating two crystallization events at different temperatures. Clearly, this phenomenon suggests a rather heterogenous phase structure formed during cooling. Starting from 180 °C in the molten state, one can envisage a rather homogenous melt structure with all co-units distributed homogenously in the melt. Upon crystallization during cooling at around 110 °C, long ethylene segments solidify, forming thick crystals, while co-units containing chain segments segregate forming regions crystallized at lower temperature. The effect of such double peak crystallization during cooling can also be seen in the heating curve where a broad melting temperature range is observed. Both the DSC cooling and heating results suggest a crystallization induced phase separation of the system forming a non-uniform distribution of co-units in the sample. 

**Figure 2:**
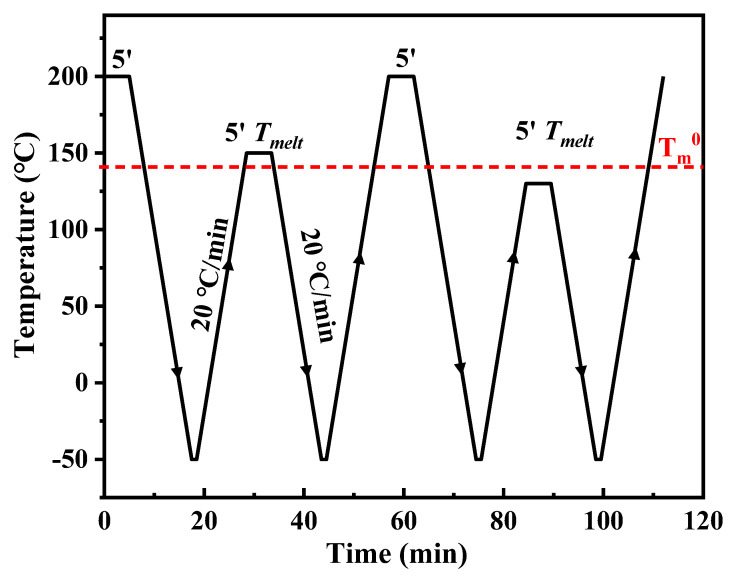
DSC thermal protocol for melting and cooling runs at 20 °C/min. The horizontal line corresponds to the equilibrium melting temperature ($T_{m}^{0}$) of the polyethylene terpolymer.

To investigate the effect of such heterogeneity on the crystallization behavior of the system, we first used a thermal protocol shown in Figure 2, according to the literature [23,30]. The sample was heated to 200 °C and kept for 5 min to ensure a complete erasure of the preceding thermal history. Then, the melts were cooled down to −50 °C and heated up to the temperature, which was denoted as *T_melt_* for 5 min followed by another cooling run to measure the crystallization behavior of the system experienced this specific *T_melt_*. The crystallization temperatures were recorded from the main peak of the cooling exotherms. As shown in the thermal protocol of Figure 2, *T_melt_* for each cooling trial can be above and below the equilibrium melting temperature. 

**Figure 3:**
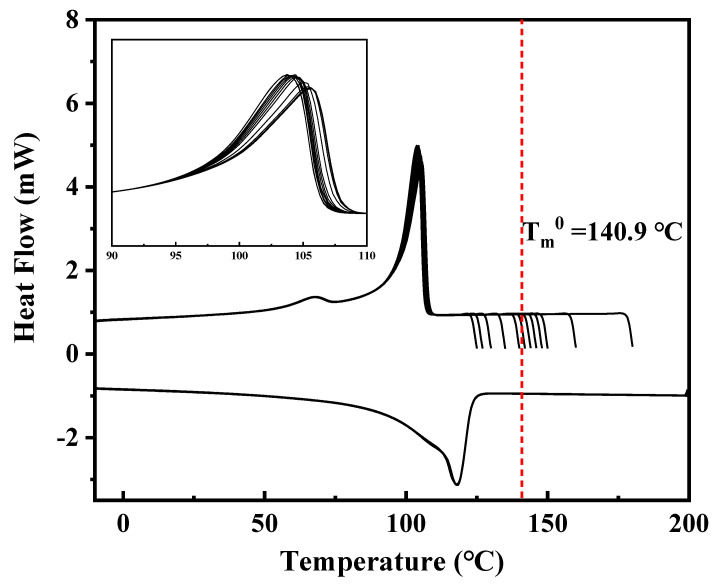
Plots of heat flow against temperature for cooling from melt temperatures in a range of 125 to 180 °C and subsequent heating at 20 °C/min for PE. The dashed line is drawn at the equilibrium melting of the copolymers, $T_{m}^{0}=140.9{}^{\circ}C$. Exothermic peaks point up, and endothermic peaks point down.

Figure 3 shows the DSC cooling and heating curves of the sample after being treated at different *T_melt_*. First, one finds a nearly completely overlapping of the melting curves of the sample being cooled down from different melt temperature, suggesting that the final structure of the sample is nearly constant regardless of the pre-treatments. The second obvious feature of the results is that the weak crystallization peak located around 70 °C remains also unchanged when *T_melt_* is changed. This result indicates that the microstructure, as well as the crystallization habit of the weak crystallizable fractions, are independent of the main crystallization process. The main crystallization peak around 110 °C showed certain *T_melt_* dependency, as also presented in the enlarged figure in the inset. This is a typical melt memory effect of crystallization often observed in copolymers. Clearly, with the increase in *T_melt_*, the main peak crystallization temperature (*T_c_*) decreases gradually. Eventually, there exists a temperature above which *T_c_* stays the same. 

**Figure 4:**
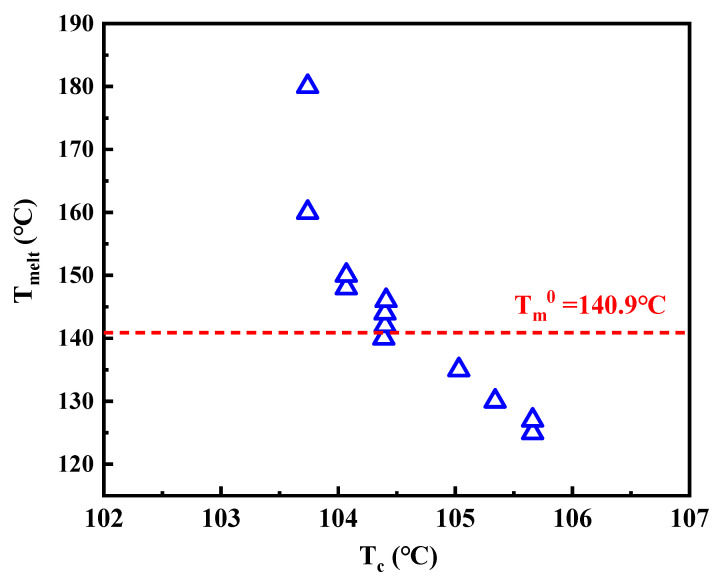
The melt temperature (*T_melt_*)-dependent peak crystallization temperature (*T_c_*) for long linear polyethylene sequences. The crystallization temperatures were recorded from the peak of the cooling exotherms.

In Figure 4, we plotted the peak crystallization temperature *T_c_* as a function of *T_melt_*. As was discussed, when the *T_melt_* was above 150 °C, *T_c_* remains constant, suggesting a homogenous melt structure before cooling down. With the decrease in *T_melt_*, a gradual increase in *T_c_* is observed, indicating a more heterogenous melt structure that can initiate nucleation, which was preserved when the melt temperature was not high enough. Clearly, when the melt temperature is low, typically below the equilibrium melting temperature of the polyethylene crystals, there might be unmolten crystals that serve as nuclei during cooling, increasing the crystallization temperature. The number of such nuclei decreases with the increasing the melt temperature. When the melting temperature is higher than the equilibrium melting temperature of polyethylene, the observed melt memory effect cannot be attributed to such un-molten crystals. There must be other types of melt structural heterogeneities that lead to nucleation at higher temperatures during cooling. Recalling the crystallization peak at around 70 °C presented in DSC cooling curves, we may consider a crystallization-induced phase separated structure with co-units enrichment within one phase. Such a co-unit-rich phase forms after the main crystallization, and it further crystallizes at around 70 °C. Obviously, the structure and composition of the co-units rich phase is not very much affected by the main crystallization temperature. It always ends up with a same microstructure so that the same weak crystallization at the same temperature during cooling. Upon heating, this phase separated structure requires higher temperature to become homogenous, which is higher than the equilibrium temperature of the crystals. It must been mentioned that the corresponding linear polyethylene without any co-units with similar molecular weight does not show such melt memory effect as soon as the melt temperature is higher as compared to the end of nominal melting in DSC curves. This is because of the fast relaxation/diffusion of the polymer chains in the molted state, producing homogenous melt, which eliminated previous thermal history completely. For comparison, the readers are directed to results presented in Figure 15 of ref. [23].

Data obtained during non-isothermal processes presented in Figure 4 showed a clear crystallization memory effect, but with rather less detailed information. To further investigate how the heterogenous melt structure due to the small fraction of co-units within the system affects the crystallization habit of the sample, kinetics of isothermal crystallization at different temperatures after the sample was processed at different melt temperatures was carried out.

**Figure 5:**
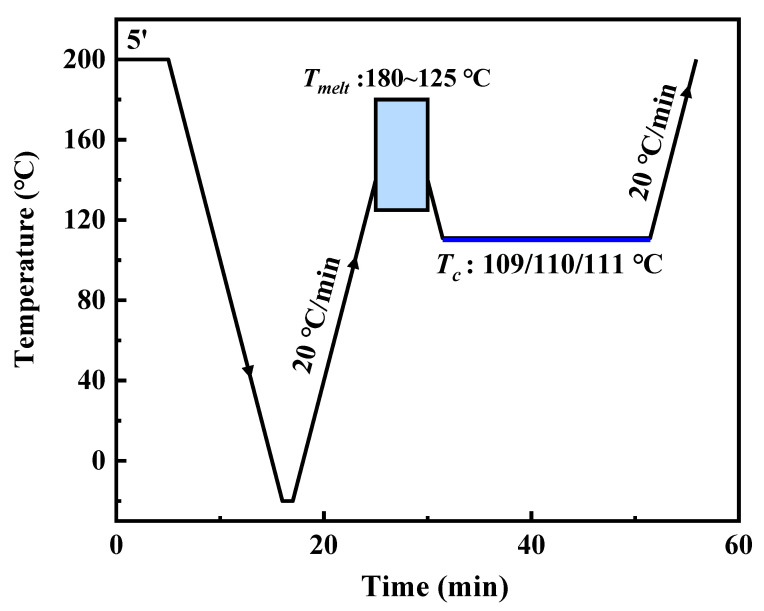
DSC thermal protocol with different melt temperatures (*T_melt_*) and different isothermal crystallization temperatures (*T_c_*).

Figure 5 shows the main thermal protocol that was used for researching the kinetics of isothermal crystallization. The heating and cooling rates were 20 °C/min, respectively. The protocol consisted of two parts, which were preparation and testing. After erasing the thermal history at 200 °C for 5 min, the sample was cooled down to −20 °C to prepare a standard sample. It was then heated up to different melted temperatures from 180 to 125 °C. Then, the melts were cooled down to the isothermal crystallization temperature (*T_c_*) to allow a full crystallization. Here, three isothermal crystallization temperatures (109, 110, and 111 °C) were chosen. 

**Figure 6:**
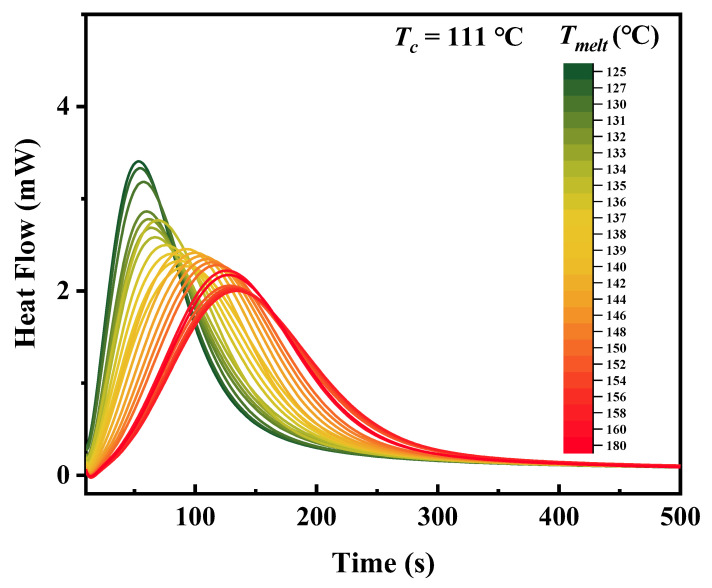
The isothermal crystallization curves of *T_c_* = 111 °C at different melt temperatures (*T_melt_*) from 125 to 180 °C.

Figure 6 presents the DSC isothermal curves at 111 °C after being treated at different melt temperatures. The heat flow curves as a function of time clearly present kinetics of isothermal crystallization at 110 °C. A change in melt temperature has a strong effect on the kinetics of isothermal crystallization temperature. Besides, the shape of the curves changes, too, for samples treated at different melt temperatures. There is nearly no induction time for samples treated at lower melt temperatures, meaning a much stronger heterogenous nucleation effect. For samples treated at higher melt temperature, the curves are rather broad, with a short induction time, suggesting a much smaller number of nuclei in the system at such cases. 

**Figure 7:**
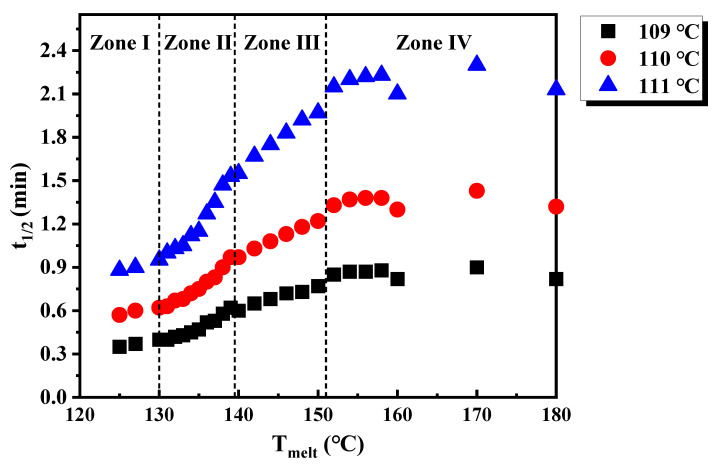
The Half crystallization times (t_1/2_) of isothermal crystallization at 109, 110, and 111 °C as a function of the melt temperatures (*T_melt_*) from 125 to 180 °C.

The peak position of the DSC isothermal curves can be used at a characteristic time for isothermal crystallization, namely, the half crystallization time (t_1/2_). t_1/2_ of samples isothermally crystallized at three temperatures after being treated at different melt temperatures are collected in Figure 7. First of all, one observes a clear overall increase in the half crystallization time at higher isothermal crystallization temperature as expected. Secondly, half crystallization time at all three isothermal crystallization temperatures shows similar melt temperature dependency. Most interestingly, several zones of melt temperature can be recognized according to the change in half crystallization time as a function of melt temperature. Briefly, zone I, form melt temperature of 125 to about 130 °C, the half crystallization time remains very short and increases only slightly; Zone II, from 130 to about 140 °C, the half crystallization time increases with the melt temperature rapidly; Zone III, from 140 to about 152 °C, the half crystallization time increases continuously with the increase in melt temperature but with a smaller slope; Zone IV, for melt temperature higher than 152 °C, the half crystallization time is essentially unchanged with the increase in melt temperature.

Understanding of the change in isothermal crystallization kinetics due to melt temperature requires knowledge of the microstructure of the system, as indicated by the DSC heating and cooling results presented in Figure 1. The observed shortest half crystallization time in zone I is clearly due to the incomplete melting of the previous crystals that serve as nuclei during isothermal crystallization. In zone II, as the melt temperature is still below the equilibrium melting temperature of the polyethylene crystals, there might be still unmolten crystals serving as nuclei during followed crystallization, or there might be residual ordering of chain segments that promotes nucleation later. With the current experimental technique, it is not possible to rule out either possibility. However, for zone III, it is certain that all crystals must be removed, as the melt temperature was higher than the equilibrium melting temperature of polyethylene crystals. The observed strong change in half crystallization time in zone III can thus only be related to the melt structure. As discussed before, phase separation induced by the main crystallization push co-units into a region that can only crystallize at around 70 °C. These low-temperature crystallized regions would undergo melting earlier than those crystals formed during main crystallization at around 110 °C. However, homogenization of such a phase separated structure requires much higher temperature. From the results shown in Figure 7, we concluded that a homogenous melt structure can be achieved at a melt temperature of about 152 °C. Therefore, in zone IV, the half crystallization time is independent of melt temperature.

## 4. Conclusions

A polyethylene random terpolymer with small amount of 1-octene and 1-hexene has been prepared, and its melt memory effect during crystallization has been investigated via non-isothermal and isothermal DSC tests. It has been found that the small amount of co-unit (0.8 mol% 1-octene and 0.3 mol% 1-hexene) initiated a strong effect on the crystallization kinetics when the sample was treated at different melt temperatures. Isothermal crystallization results clearly show four zones of the melt temperature at which the characteristic features of the isothermal half crystallization time change. The four zones can be due to incomplete melting of the crystals (zone I), crystal residuals or remaining of chain segmental orientation (zone II), gradual vanishing of the phase separated melt structure (zone III), and, finally, a homogenous melt structure (zone IV). The experimental results presented in this work are considered relevant in guiding the real-life application of the material during its processing. By tuning the melt temperature, one expects a rather rich melt structures so that different melt properties can be obtained. Nevertheless, this work focused on a single sample and one aspect of crystallization only. Further works on two directions are clearly needed. Firstly, the effect of the number of co-units and their relative contains in the terpolymers should be clarified. Secondly, knowledge of the effect of such melt heterogenous structure on crystallization under shear flow is desired, which will have strong impact on processing.

## Figures and Tables

**Table 1:** Characterization of the P(E-co-O-co-H) terpolymer.

Sample	Ethylene (mol%)	Octene (mol%)	Hexene (mol%)	M_w_ (g/mol)	M_n_ (g/mol)	M_w_/M_n_	T_m_^a^ (°C)	Φ_w_^b^ (%)
P(E-co-O-co-H)	98.9	0.8	0.3	106,600	41,700	2.55	116.2	42.1

^a^ Peak temperature from DSC cooling and heating at 10 °C/min. ^b^ the melting enthalpy of polyethylene used for calculated the DSC crystallinity is 293 J/g.

## Data Availability

Data can be provided upon request.
